# Correlation of the OCT Double-Layer Sign with Type 1 Non-Exudative Neovascularization on OCT-A in Age-Related Macular Degeneration

**DOI:** 10.3390/medicina59101829

**Published:** 2023-10-14

**Authors:** Dragana Ristic, Mirko Resan, Igor Pancevski, Petar Ristic, Miroslav Vukosavljevic, Milos Cvetkovic, Bojan Pajic

**Affiliations:** 1 Eye Clinic, Military Medical Academy, 11000 Belgrade, Serbia; resan.mirko@gmail.com (M.R.); dr.panchevski@gmail.com (I.P.); miloscvetkovicvma@gmail.com (M.C.); 2 Faculty of Medicine of the Military Medical Academy, University of Defense, 11000 Belgrade, Serbia; petris011@yahoo.com (P.R.); vuki032@yahoo.com (M.V.); bojan.pajic@orasis.ch (B.P.); 3 Department of Physics, Faculty of Sciences, University of Novi Sad, 21000 Novi Sad, Serbia; 4 Endocrinology Clinic, Military Medical Academy, 11000 Belgrade, Serbia; 5 Military Medical Academy Management, 11000 Belgrade, Serbia; 6 Division of Ophthalmology, Department of Clinical Neurosciences, Geneva University Hospitals, 1205 Geneva, Switzerland; 7 Experimental Ophthalmology, University of Geneva, 1205 Geneva, Switzerland; 8 Eye Clinic ORASIS, Swiss Eye Research Foundation, 5734 Reinach, Switzerland

**Keywords:** retina, OCT, OCT-A, neovascular membrane, double-layer sign, choroidal neovascularization, age-related macular degeneration

## Abstract

*Background and Objectives*: Early diagnosis of the exudative form of age-related macular degeneration (AMD) is very important for a timely first treatment, which is directly related to the preservation of functional visual acuity over a long period. The goal of this paper was to examine the correlation between the double-layer sign (DLS) and the presence of non-exudative macular neovascularization (MNV). *Materials and Methods*: Our research included 60 patients with AMD, exudative in one eye and non-exudative in the other eye. We analyzed only the non-exudative form using optical coherence tomography (OCT) and optical coherence tomography angiography (OCT-A). The patients were classified into three groups, depending on the duration of the disease (<2 years, 2 to 5 years, >5 years). The onset of the disease was deemed the moment of establishing a diagnosis of exudative AMD in one eye. We defined the presence or absence of a DLS using OCT and the presence of non-exudative MNV using OCT-A, both on 3 × 3 mm and 6 × 6 mm sections. DLS was used as a projection biomarker for non-exudative MNV, with the aim of establishing a rapid diagnosis and achieving early treatment of the disease. *Results*: We found that there was a statistically significant correlation between the DLS diagnosed using OCT and non-exudative MNV diagnosed by OCT-A for both 3 × 3 mm (*p* < 0.001) and 6 × 6 mm (*p* < 0.001) imaging. There was a statistically significant difference between the frequencies of both DLS and MNV in Groups I and III on both 3 × 3 and 6 × 6 mm imaging. A statistically significant difference was also noted in the frequencies of DLS and MNV on 6 × 6 mm imaging, but not on 3 × 3 mm imaging, between Groups I and II. No differences were found between the frequencies of DLS and MNV between Groups II and III. *Conclusions*: The DLS on OCT can be used as a projection biomarker to assess the presence of a non-exudative MNV.

## 1. Introduction

Age-related macular degeneration (AMD) is one of the leading causes of blindness in developed countries, particularly in people older than 60 years [1]. The disease progresses over time, so the final stage includes the presence of geographic atrophy or the emergence of neovascularization [2]. The introduction of anti-VEGF (vascular endothelial growth factor) therapy in the treatment of neovascular AMD (nAMD) represented a milestone in preserving visual acuity in these patients [3,4,5]. In order to have as good an effect of the applied therapy as possible, it is vital to recognize the active membrane early and start the treatment. With patients who already suffer from a neovascular membrane in one eye, it is important to also observe the other eye, especially since the risk of occurrence of macular neovascularization (MNV) in the other eye is deemed 42% within a period of 5 years following the occurrence of MNV in one eye [6].

Macular neovascularization is typically associated with the occurrence of exudation and degradation of visual acuity. However, new diagnostic procedures have allowed for the detection of non-exudative MNV as well. This form is characterized by the absence of exudation and occurs in patients who are mostly asymptomatic [7,8,9]. Non-exudative MNV most often refers to type 1 MNV, although other categories of neovascular membranes can also be without exudation [10]. The significance of the non-exudative membrane is reflected in the fact that the risk of exudation is higher, which makes intensive observation of these patients significant [11].

The most relevant procedures for diagnosing nAMD are optical coherence tomography (OCT), optical coherence tomography angiography (OCT-A), fluorescein angiography (FA), and indocyanine green angiography (ICGA) in the case of polypoidal choroidal vasculopathy. In exudative MNV, the presence of fluid in the macula can be clearly seen on the OCT, as opposed to the non-exudative form with no visible fluid. With these patients, MNV can be visualized using OCT-A [12,13,14,15,16,17,18].

**Figure 1:**
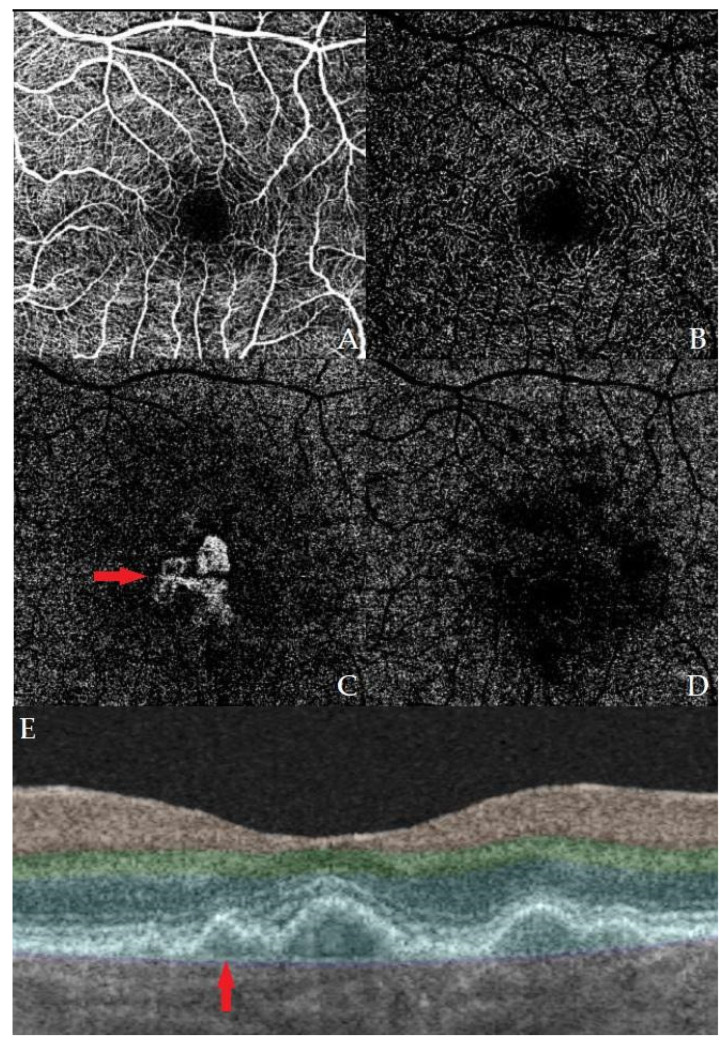
Example images of OCT and OCT-A of one of our patients with a macular neovascular membrane. Images (**A**–**D**) represent OCT-A of different layers of the macular vascular network, specifically (**A**) superficial, (**B**) deep, (**C**) outer retina, and (**D**) choriocapillaris. A neovascular membrane can be seen in image (**C**). Image (**E**) shows an OCT B-scan of the macula with a present DLS.

In 2013, Querques et al. made the first description of non-exudative MNV using OCT and ICGA (indocyanine green angiography) [8]. The presence of two hyperreflective layers on OCT (RPE and Bruch’s membrane) or a shallow, irregular ablation of the RPE is called the “double-layer sign” (DLS) (Figure 1). This term was first introduced by Sato et al., and it was described in the presence of vascular network branching in polypoidal choroidal vasculopathy [19]. In 2019, Shi et al. showed that the DLS detected by the OCT was correlated with non-exudative MNV [20].

Making a correlation between the DLS as an OCT biomarker of present non-exudative MNV and the presence of a neovascular membrane finding diagnosed by the OCT-A method would speed up the diagnosis and, consequently, the period of treatment for these patients. Furthermore, in developing countries or small regional centers, there may be a lack of access to OCT-A, and recognition of the DLS on OCT and prompt referral may make a difference in treatment outcomes.

In summary, macular neovascularization in AMD can be present without overlying fluid, and this neovascular membrane can be detected with OCT-A even if inactive. The presence of a double linear sign can be correlated with the existence of a non-exudative macular neovascular membrane.

The goal of this study is to examine the correlation between the DLS detected using OCT and the presence of non-exudative MNV through OCT-A in patients who have already been diagnosed with MNV in one eye.

## 2. Materials and Methods

A retrospective study was conducted at the Eye Clinic of the Military Medical Academy in Belgrade. The study was approved by the Ethics committee of the Military Medical Academy. The study included patients with exudative AMD in one eye and non-exudative AMD in the other who underwent examinations at the Military Medical Academy from October 2022 to June 2023 and whose medical records including OCT and OCT-A images were stored. We analyzed only the eyes with the non-exudative AMD. We analyzed the data of 60 patients (60 eyes). We classified the participants into three groups (20 participants per group) according to the onset of the disease. The onset of the disease was deemed at the moment when the diagnosis of exudative AMD in one eye had been established. The first group consisted of participants suffering from the disease for less than two years, the second group consisted of participants suffering from the disease for two to five years, and the third group consisted of participants suffering from the disease longer than five years. Exclusion criteria included any other diseases of the posterior segment and opacified media. Moreover, the study included patients to whom anti-VEGF therapy was administered because of active MNV in one eye, but only if the last administered dose was three months prior to the OCT and OCT-A imaging in order to exclude any possible effects on the other eye.

All eyes in the study underwent SS (swept-source) OCT and SS OCT-A imaging (Triton DR 1000; Topcon, Tokyo, Japan). This device uses a central wavelength of 1.050 nm and a scanning speed of 100,000 A-scans per second. The acquired scans are displayed simultaneously as separate “en face” images of the retinal microvasculature and choriocapillaris. It is worth noting that the OCTARA (OCT-A ratio analysis) algorithm generates OCT-A images by registering a B-scan repetition at each scan location and then computing a ratio-based result between corresponding image pixels. This method preserves the integrity of the OCT spectrum and does not result in compromised axial resolution, an inherent disadvantage of other OCT-A technologies [21].

The absence of macular fluid was determined after a review of the retinal thickness maps and B-scans from the SS OCT images. After the B-scans, each eye was evaluated for the presence or absence of a DLS by two independent graders (authors D.R. and M.R.).

All eyes in this study were imaged using the 3 × 3 mm and 6 × 6 mm scan patterns, so we observed not only different-sized areas of the macula but also different qualities of the images. All scans were centered on the fovea, and the SMARTTrack motion correction software (Topcon, Tokyo, Japan) was used during image acquisition. Non-exudative MNV not fully contained or outside the 6 × 6 mm field of view were excluded from the study.

For the visualization of MNV, the boundaries we used in this study extended from the outer retina to the choriocapillaris. No segmentation was manually altered for this study.

Data analysis was conducted in Python 3 using the Anaconda distribution and included libraries. Because nominal data were collected, chi-squared or Fisher tests were used, depending on the sample size. Sensitivity, specificity, and accuracy values were calculated using their respective formulas.

## 3. Results

We analyzed the data of 60 participants split into three groups (20 participants per group). The age ranged between 62 and 80 years (average of 73.6 years in the first, 73.9 in the second, and 74.8 in the third group).

The presence or absence of DLS was analyzed on the B-scan obtained using OCT, after which we compared the presence of MNV using the OCT-A method, namely in the 3 × 3 mm and 6 × 6 mm sections (Table 1).

Table 1 shows that the first group of patients recorded no presence of DLS or non-exudative MNV on the 3 × 3 mm section. Nevertheless, seven patients within the same group had a positive DLS on a 6 × 6 mm section, while no patients had non-exudative MNV. In Group II, on the 3 × 3 section, DLS was present in three patients, in which non-exudative MNV was also recorded. On the 6 × 6 mm section, DLS was present in 15 patients, whereas 11 patients had non-exudative MNV. In Group III, both DLS and non-exudative MNV were present in 7 patients on the 3 × 3 section; DLS was present in 15 patients on the 6 × 6 section, while non-exudative MNV was also noted in 12 patients.

We analyzed the distribution of the presence of DLS and non-exudative MNV on OCT-A sections of 3 × 3 mm and 6 × 6 mm between each of the three groups separately (Fisher’s exact test), i.e., every group according to the duration of the disease was tested against the other groups. The results can be seen in Table 2.

In Table 2, we can see that there is a statistically significant difference in the presence of DLS and MNV on both 3 × 3 and 6 × 6 sections between Group I and Group III. A statistically significant difference in the presence of MNV and DLS was also noted on 6 × 6 imaging between Groups I and II. No differences were found between Groups II and III.

We used Fisher’s exact test to analyze the correlation between the DLS and non-exudative MNV on a 3 × 3 mm field, as well as DLS and MNV on a 6 × 6 mm field for all patients from the three groups combined. Values for sensitivity, specificity, and accuracy were obtained according to their standard formulas. These results can be seen in Table 3.

We observed a statistically significant correlation between DLS and non-exudative MNV on both the 3 × 3 mm images and 6 × 6 mm images. On the 3 × 3 images, the DLS was a perfect biomarker of MNV according to our results, having an accuracy of 100%. For the 6 × 6 images, while a high sensitivity was noted (100%), the specificity of DLS was only 62.2% because of the emergence of false positive results (eyes in which a DLS was recorded but not MNV). There were no false negative results, meaning no patients had MNV on OCT-A without the presence of a DLS.

## 4. Discussion

Treatment of the exudative form of AMD through the administration of anti-VEGF therapy was introduced almost 20 years ago [4,22,23]. In order to reach the best possible therapeutic outcomes, it is important to recognize exudative MNV in time and start treatment as soon as feasible. Considering the fact that the disease progression over a time span of five years, from the early or intermediate stage to the late stage, ranges from 0.4% to as much as 53%, depending on the stage of the disease, it is obvious how important it is to find predictive biomarkers that can help us in early diagnosis of non-exudative MNV [24]. Modern diagnostic procedures, such as OCT and OCT-A, are of great significance for reaching the rapid diagnosis of MNV by defining specific biomarkers for the detection of non-exudative MNV and observing its progression. One such biomarker that can be deemed the golden projection standard in the detection of non-exudative MNV is DLS obtained using the OCT method [24].

Yang et al. described that the emergence of non-exudative MNV in one eye in patients in whom exudative MNV had been diagnosed in the other eye equaled 13.2%, which was higher than findings by Paleywala et al., which had equaled 6.25% [11,25]. Moreover, Yang et al. stated in their study that the risk of exudation increased with the extending duration of the disease (from 24% after one year of observing to 34.5% after two years of observing), which is why we also divided our patients into three groups depending on the duration of the disease and found that the frequency of occurrence of a non-exudative MNV increased with the extension of the disease duration, which can be followed up by an increase in the frequency of DLS with patients whose disease lasted longer.

In our study, we observed various stages of AMD, primarily related to the disease duration, and we compared the finding of DLS on OCT (presence or absence) with the finding of non-exudative MNV diagnosed using the OCT-A method (presence or absence).

OCT-A is the only diagnostic procedure that is capable of diagnosing MNV even when fluid is absent on OCT or on fluorescein angiography (FA) [12,25].

The diagnosis of non-exudative MNV increased significantly by using OCT-A. Laiginhas et al. described this in their paper and stated that it ranged from 6.25% to 27% in patients who suffered from exudative AMD in the other eye [26]. Further on, we found in our study that the number of patients with non-exudative MNV was higher in Groups II and III relative to the first group, where we did not have a single non-exudative MNV.

If we compare the risk of transition from non-exudative to exudative MNV, such risk ranges from 21.1% with patients diagnosed with exudative AMD in one eye to 5.4% with patients with no exudative AMD in either eye [8,25,26]. This is precisely why we decided to observe patients who already suffered from an exudative form of AMD in one eye.

Analysis of earlier OCT-A studies showed that non-exudative type 1 MNV correlates with DLS on OCT B-scan [11,14,15,16,17,18]. This correlation, which we also observed in our study, is very significant because it speaks in favor of the prognostic role of DLS in the diagnosis of non-exudative MNV. This was also a topic of the study written by Shi et al. [20].

We applied the OCT-A as the golden standard for the detection of non-exudative MNV in our study and then compared this finding with the presence of DLS found through OCT. Our results showed that not all eyes where we found DLS suffered from the non-exudative MNV as well. In the first group of patients, with a disease period of less than two years, we found no DLS or non-exudative MNV on 3 × 3 mm images. However, for the 6 × 6 mm images, we detected a number of false positives, the reason for the drop in specificity for the 6 × 6 mm fields. We would also expect a drop in sensitivity and specificity values in larger sample sizes since, for certain groups in our research, no false positives or false negatives were found, which is something that we would expect to find.

Why did we get different values for 3 × 3 mm relative to 6 × 6 mm imaging? Despite the known fact that 3 × 3 mm scans cover smaller area of the macula, the resolution is better than with 6 × 6 mm OCT-A scans. The difficulty in enlarging the field without sacrificing resolution is a significant issue for the development of OCT-A technology, as its field of view is significantly smaller than modalities such as FA [27]. It is possible that a worse resolution might have been a reason for our inability to recognize non-exudative MNV.

Min Gao et al. also commented that poor segmentation leads to errors in the corresponding en face OCT-A images with false positive or negative flow signals. They suggested that, in these situations, a manual adjustment of the layer segmentation should be performed to optimize OCT-A scans to aid clinical interpretation [27].

Shi et al., Narita et al., and Ghanci et al. showed that DLS of high sensitivity (83–100%) and specificity (74–94%) correlates with the presence of non-exudative MNV [20,28,29]. In our paper, we obtained sensitivity, specificity, and accuracy for DLS as a marker for non-exudative MNV on 3 × 3 mm images of 100%, while the specificity and accuracy differed on 6 × 6 mm images and equaled 62.2% and 76.6%, respectively, with sensitivity remaining at a 100%. A high sensitivity for DLS as a sign of MNV makes it a very good screening test in situations where OCT-A or FA is unavailable.

It is also worth noting that some authors have noted different types of DLS, specifically a “thick” and “thin” DLS, and studies have shown that a thick DLS specifically was a strong predictor for exudative MNV, while a thin DLS was deemed not to be a risk factor [30]. No such distinction was made in this study.

In our study, patients with present DLS but absent non-exudative MNV do not represent a significant portion, but it could represent a problem in everyday practice. This research would be most significant for regional ophthalmological centers where there is no access to OCT-A, but there is access to OCT. Recognition of the DLS can be a reason for prompt referral to a tertiary center where OCT-A is available and thus may result in better treatment outcomes because of early recognition of MNV, something that might not be otherwise possible.

A large number of authors have tried to find characteristic parameters that can predict the transition from non-exudative to exudative MNV. In the existing literature, one can see that approximately 25% of non-exudative MNV translates into exudative in a period of 6 to 20 months [26].

Relying upon the data that showed that, in a period of 6–8 months, there was a high risk for the occurrence of exudation in the presence of non-exudative MNV, Invernizzi et al. [31] suggested a shorter period between check-ups.

Additional studies are needed to examine whether other biomarkers can be included, which, together with the DLS, can help in the rapid diagnosis of non-exudative MNV and/or predict its transition to the exudative form.

One of the limitations of our study was our inability to track these patients until their likely transition into exudative AMD, and thus, we were unable to follow up on the emergence of exudation and the OCT findings related to it. Another limitation is the small sample size in each group due to a large number of patients being excluded because of either having exudative AMD in both eyes or because they received anti-VEGF therapy less than 3 months before the initial OCT and OCT-AA imaging. Another limitation of the study that is also important to note is that, even though it is used as a gold standard test in this study, previous studies have shown that OCT-A is not capable of detecting all NVM and that the addition of FA as a diagnostic tool together with OCT has shown better results at detecting neovascularization than OCT-A alone [32]. Inoue et al. showed in their paper that factors that correlate with the sensitivity of OCT-A are the signal strength, the height of PEDs, and previous treatment. In this study, no pre-selected cut-off value for the signal strength was used. However, since these scans were performed as a part of regular patient examination, it was left to the discretion of the examiner to repeat the scan if needed, whether that was due to improper segmentation or inadequate signal strength.

## 5. Conclusions

In summary, we found that the double-layer sign on structural OCT B-scans could predict the presence of subclinical MNV in most eyes with non-exudative AMD identified through SS OCT-A imaging with a high sensitivity.

By combining DLS as an OCT biomarker and observing the activity of non-exudative MNV through OCT-A, we can recognize MNV and follow up with patients on a more frequent basis or refer them to an appropriate tertiary institution in order to start anti-VEGF treatment in due time, owing to the fact that the MNV exudation is detected earlier, preserving the functional visual acuity of the patients for as long as feasible. However, we expect the greatest benefit from this in community hospitals and in smaller regional centers where only OCT, and not OCT-A, is available.

Considering, on the one hand, the risk of loss of central visual acuity and the quality of life and, on the other hand, the benefit of anti-VEGF therapy, especially if commenced in time, our recommendation based on our findings and existing literature is to perform OCT-A on patients with a DLS on OCT, and for those patients, we recommend more frequent check-ups and self-control at home by using an Amsler grid.

## Figures and Tables

**Table 1:** Frequencies of DLS and MNV detection in Groups I, II, and III on 3 × 3 and 6 × 6 mm OCT and OCT-A imaging.

Groups	Group I		Group II		Group III	
Parameters	OCT-A 3 × 3	OCT-A 6 × 6	OCT-A 3 × 3	OCT-A 6 × 6	OCT-A 3 × 3	OCT-A 6 × 6
DLS present DLS absent MNV present MNV absent	0 (0%) 20 (100%) 0 (0%) 20 (100%)	7 (35%) 13 (65%) 0 (0%) 20 (100%)	3 (15%) 17 (85%) 3 (15%) 17 (85%)	15 (75%) 5 (25%) 11 (55%) 9 (45%)	7 (35%) 13 (65%) 7 (35%) 13 (65%)	15 (75%) 5 (25%) 11 (55%) 9 (45%)

**Table 2:** Comparisons between the frequencies of MNV and DLS on 3 × 3 and 6 × 6 mm images on OCT and OCT-A for each group. The table contains the *p*-values from Fisher tests; statistically significant values are in bold. Frequencies and percentages for each of the groups can be seen in Table 1.

Variable	MNV 3 × 3	DLS 3 × 3	MNV 6 × 6	DLS 6 × 6
Group I vs. Group II	*p* = 0.231	*p* = 0.231	***p* < 0.001**	***p* = 0.025**
Group II vs. Group III	*p* = 0.237	*p* = 0.237	*p* = 1	*p* = 1
Group I vs. Group III	***p* = 0.008**	***p* = 0.008**	***p* < 0.001**	***p* = 0.002**

**Table 3:** Values for sensitivity, specificity, and accuracy for DLS as a predictive marker for MNV on 3 × 3 and 6 × 6 mm images, as well as *p*-values for chi-square tests for correlation.

	DLS for MNV on 3 × 3 mm	DLS for MNV on 6 × 6 mm
Sensitivity	100%	100%
Specificity	100%	62.2%
Accuracy	100%	76.6%
*p*-value	<0.001	<0.001

## Data Availability

The data presented in this study are available on request from the corresponding author. The data are not publicly available because they contain confidential patient information.

## References

[1] Wong W.L., Su X., Li X., Cheung C.M.G., Klein R., Cheng C.Y., Wong T.Y. (2014). Global prevalence of age-related macular degeneration and disease burden projection for 2020 and 2040: A systematic review and meta-analysis. Lancet Glob. Health.

[2] Holz F.G., Schmitz-Valckenberg S., Fleckenstein M. (2014). Recent developments in the treatment of age-related macular degeneration. J. Clin. Investig..

[3] Michels S., Rosenfeld P.J., Puliafito C.A., Marcus E.N., Venkatraman A.S. (2005). Systemic bevacizumab (Avastin) therapy for neovascular age-related macular degeneration twelve-week results of an uncontrolled open-label clinical study. Ophthalmology.

[4] Rosenfeld P.J., Moshfeghi A.A., Puliafito C.A. (2005). Optical Coherence Tomography Findings After an Intravitreal Injection of Bevacizumab (Avastin^®^) for Neovascular Age-Related Macular Degeneration. Ophthalmic Surg. Lasers Imaging Retina.

[5] Ristić D., Vukosavljević M., Draganić B., Cerović V., Petrović N., Janićijević-Petrović M. (2013). The effect of intravitreal administration of bevacizumab on macular edema and visual acuity in age-related macular degeneration with subfoveolar choroidal neovascularisation. Vojnosanit. Pregl..

[6] Maguire M.G., Bressler S.B., Bresskr N.M., Alexander J., Hiner C.J., Javomik N.B., Phillips D., Marsh M., Haivfeins B.S., Burgess D.B. (1997). Risk factors for choroidal neovascularization in the second eye of patients with juxtafoveal or subfoveal choroidal neovascularization secondary to age-related macular degeneration. Arch. Ophthalmol..

[7] Dansingani K.K., Freund K.B. (2015). Optical Coherence Tomography Angiography Reveals Mature, Tangled Vascular Networks in Eyes With Neovascular Age-Related Macular Degeneration Showing Resistance to Geographic Atrophy. Ophthalm. Surg. Lasers Imag. Retina.

[8] Querques G., Srour M., Massamba N., Georges A., Ben Moussa N., Rafaeli O., Souied E.H. (2013). Functional characterization and multimodal imaging of treatment-naïve “quiescent” choroidal neovascularization. Investig. Ophthalmol. Vis. Sci..

[9] Chen L., Messinger J.D., Sloan K.R., Swain T.A., Sugiura Y., Yannuzzi L.A., Curcio C.A., Freund K.B. (2020). Nonexudative macular neovascularization supporting outer retina in age-related macular degeneration: A clinicopathologic correlation. Ophthalmology.

[10] Sacconi R., Fragiotta S., Sarraf D., Sadda S.R., Freund K.B., Parravano M., Corradetti G., Cabral D., Capuano V., Miere A. (2023). Towards a better understanding of non-exudative choroidal and macular neovascularization. Prog. Retin. Eye Res..

[11] De Oliveira Dias J.R., Zhang Q., Garcia J.M.B., Zheng F., Motulsky E.H., Roisman L., Miller A., Chen C.L., Kubach S., de Sisternes L. (2018). Natural history of subclinical neovascularization in nonexudative age-related macular degeneration using swept-source OCT angiography. Ophthalmology.

[12] Yanagi Y., Mohla A., Lee W.-K., Lee S.Y., Mathur R., Chan C.M., Yeo I., Wong T.Y., Cheung C.M.G. (2017). Prevalence and Risk Factors for Nonexudative Neovascularization in Fellow Eyes of Patients With Unilateral Age-Related Macular Degeneration and Polypoidal Choroidal Vasculopathy. Investig. Opthalmol. Vis. Sci..

[13] Palejwala N.V., Jia Y., Gao S.S., Liu L., Flaxel C.J., Hwang T.S., Lauer A.K., Wilson D.J., Huang D., Bailey S.T. (2015). Detection of nonexudative choroidal neovascularization in age-related macular degeneration with optical coherence tomography angiography. Retina.

[14] Roisman L., Zhang Q., Wang R.K., Gregori G., Zhang A., Chen C.-L., Durbin M.K., An L., Stetson P.F., Robbins G. (2016). Optical Coherence Tomography Angiography of Asymptomatic Neovascularization in Intermediate Age-Related Macular Degeneration. Ophthalmology.

[15] Novais E.A., Adhi M., Moult E.M., Louzada R.N., Cole E.D., Husvogt L., Lee B., Dang S., Regatieri C.V., Witkin A.J. (2016). Choroidal Neovascularization Analyzed on Ultrahigh-Speed Swept-Source Optical Coherence Tomography Angiography Compared to Spectral-Domain Optical Coherence Tomography Angiography. Am. J. Ophthalmol..

[16] Lane M., Moult E.M., Novais E.A., Louzada R.N., Cole E.D., Lee B., Husvogt L., Keane P.A., Denniston A.K., Witkin A.J. (2016). Visualizing the Choriocapillaris Under Drusen: Comparing 1050-nm Swept-Source Versus 840-nm Spectral-Domain Optical Coherence Tomography Angiography. Investig. Opthalmol. Vis. Sci..

[17] Moult E., Choi W., Waheed N.K., Adhi M., Lee B., Lu C.D., Jayaraman V., Potsaid B., Rosenfeld P.J., Duker J.S. (2014). Ultrahigh-Speed Swept-Source OCT Angiography in Exudative AMD. Ophthalmic Surg. Lasers Imaging Retina.

[18] Zhang A., Zhang Q., Wang R.K. (2015). Minimizing projection artifacts for accurate presentation of choroidal neovascularization in OCT micro-angiography. Biomed. Opt. Express.

[19] Sato T., Kishi S., Watanabe G., Matsumoto H., Mukai R. (2007). Tomographic features of branching vascular networks in polypoidal choroidal vasculopathy. Retina.

[20] Shi Y., Motulsky E.H., Goldhardt R., Zohar Y., Thulliez M., Feuer W., Gregori G., Rosenfeld P.J. (2019). Predictive Value of the OCT Double-Layer Sign for Identifying Subclinical Neovascularization in Age-Related Macular Degeneration. Ophthalmol. Retina.

[21] Moussa M., Leila M., Khalid H. (2017). Imaging choroidal neovascular membrane using en face swept-source optical coherence tomography angiography. Clin. Ophthalmol..

[22] Avery R.L., Pieramici D.J., Rabena M.D., Castellarin A.A., Nasir M.A., Giust M.J. (2006). Intravitreal Bevacizumab (Avastin) for Neovascular Age-Related Macular Degeneration. Ophthalmology.

[23] Bashshur Z.F., Bazarbachi A., Schakal A., Haddad Z.A., El Haibi C.P., Noureddin B.N. (2006). Intravitreal Bevacizumab for the Management of Choroidal Neovascularization in Age-related Macular Degeneration. Am. J. Ophthalmol..

[24] Flores R., Carneiro Â., Tenreiro S., Seabra M.C. (2021). Retinal Progression Biomarkers of Early and Intermediate Age-Related Macular Degeneration. Life.

[25] Yang J., Zhang Q., Motulsky E.H., Thulliez M., Shi Y., Lyu C., de Sisternes L., Durbin M.K., Feuer W., Wang R.K. (2019). Two-Year Risk of Exudation in Eyes with Nonexudative Age-Related Macular Degeneration and Subclinical Neovascularization Detected with Swept Source Optical Coherence Tomography Angiography. Arch. Ophthalmol..

[26] Laiginhas R., Yang J., Rosenfeld P.J., Falcao M. (2020). Nonexudative macular neovascularization—A systematic review of prevalence, natural history, and recent insights from OCT angiography. Ophthalmol. Retina.

[27] Gao M., Guo Y., Hormel T.T., Sun J., Hwang T.S., Jia Y. (2020). Reconstruction of high-resolution 6×6-mm OCT angiograms using deep learning. Biomed. Opt. Express.

[28] Narita C., Wu Z., Rosenfeld P.J., Yang J., Lyu C., Caruso E., McGuinness M., Guymer R.H. (2020). Structural OCT Signs Suggestive of Subclinical Nonexudative Macular Neovascularization in Eyes with Large Drusen. Ophthalmology.

[29] Ghanchi F.D., Fulcher C., Madanat Z., Mdanat F. (2021). Optical coherence tomography angiography for identifying choroidal neovascular membranes: A masked study in clinical practice. Eye.

[30] Wakatsuki Y., Hirabayashi K., Yu H.J., Marion K.M., Corradetti G., Wykoff C.C., Sadda S.R. (2023). Optical Coherence Tomography Biomarkers for Conversion to Exudative Neovascular Age-related Macular Degeneration. Arch. Ophthalmol..

[31] Invernizzi A., Parrulli S., Monteduro D., Cereda M.G., Nguyen V., Staurenghi G., Cheung C.M.G., Gillies M., Teo K.Y.C. (2021). Outer Retinal Layer Thickening Predicts the Onset of Exudative Neovascular Age-Related Macular Degeneration. Arch. Ophthalmol..

[32] Inoue M., Jung J.J., Balaratnasingam C., Dansingani K.K., Dhrami-Gavazi E., Suzuki M., De Carlo T.E., Shahlaee A., Klufas M.A., El Maftouhi A. (2016). A Comparison Between Optical Coherence Tomography Angiography and Fluorescein Angiography for the Imaging of Type 1 Neovascularization. Investig. Opthalmol. Vis. Sci..

